# The Toxin-Producing Ability of Fusarium Proliferatum Strains Isolated from Grain

**DOI:** 10.32607/actanaturae.27546

**Published:** 2025

**Authors:** O. P. Gavrilova, A. S. Orina, T. Yu. Gagkaeva, N. N. Gogina

**Affiliations:** All-Russian Institute of Plant Protection, St. Petersburg, 196608 Russian Federation; All-Russian Scientific Research and Technological Institute of Poultry, Sergiev Posad, 141311 Russian Federation

**Keywords:** Fusarium, phylogenetic analysis, mycotoxins, HPLC-MS/MS

## Abstract

The widespread fungus *Fusarium proliferatum *can infect numerous plant species and produce a range of mycotoxins, the amount of which can vary significantly. Twelve *F. proliferatum *sensu lato strains isolated from six wheat, four oat, and two maize grain samples were analyzed. The strains were identified through a phylogenetic analysis of nucleotide sequences derived from gene fragments of the translation elongation factor EF-1α, β-tubulin, and RNA polymerase II second subunit. The mating types of the strain were determined by allele-specific PCR. Secondary toxic metabolite production by the strains was quantified using high-performance liquid chromatography-tandem mass spectrometry (HPLC-MS/MS). All twelve *Fusarium* strains formed a distinct clade alongside the *F. proliferatum *reference strains, thereby confirming the taxonomic identification. Only one idiomorph at the MAT locus in each *F. proliferatum *strain was found, indicative of heterothallic mating. The frequency of the MAT1-1 idiomorph was double that of the MAT1-2 idiomorph. The active biosynthesis of fumonisins B1 (71–6175 mg/kg), B2 (12–2661 mg/kg), and B3 (6–588 mg/kg), significant beauvericin (64–455 mg/kg), and trace amounts of moniliformin (12–6565 μg/kg) were identified across all examined *F. proliferatum *strains.

## INTRODUCTION

Among the *Fusarium *genus, the *Fusarium fujikuroi* (FF) species complex is particularly large and serves as a prime illustration of the considerable evolution undergone by species concepts. A dataset of both morphological and molecular studies reveals the FF species complex to contain more than 60 identified species, though this figure is probably an underestimate [1]. Taxonomic resolution within the FF species complex is achieved through the integration of physiological and biochemical characteristics due to the ambiguity, instability, and limited utility of morphological traits for species delimitation. Molecular technologies have revealed the paraphyletic nature of previously characterized FF species, demonstrating morphological convergence among phylogenetically disparate taxa [2, 3, 4].

Species within the FF complex include plant pathogens, endophytes, and pathogens of humans and animals [5]. The secondary metabolites produced by these fungi exhibit structural diversity and include mycotoxins and phytohormones such as gibberellins, auxins, and cytokinins [6, 7]. A comprehensive understanding of secondary metabolite diversity within various members of the FF species complex remains elusive, with potential discrepancies even between closely related species. Distinguishing between* Fusarium *species with clarity and thoroughly characterizing their properties improves the accuracy of strain identification and expands our understanding of their biological features.

One of the most actively studied members of the FF species complex is *F. proliferatum *(Matsush.) Nirenberg ex Gerlach & Nirenberg. This is due to its ubiquitous distribution and ability to infect a wide range of plants [11], including cereals, legumes [12, 13], vegetables [14], and fruit crops [15, 16, 17]. The mani festations of diseases caused by *F. proliferatum *include wilting and rot [13, 18, 19], with asymptomatic infection also frequently observed. Similar to the closely related species *F. verticillioides *(Sacc.) Nirenberg, *F. proliferatum *is one of the most harmful pathogens for maize, causing cob and stem rots [20]. Under optimal fungal growth conditions in cereal crops, infected wheat grains may exhibit stunted growth and black germ [21], while infected oats may display discoloration, necrotic lesions on spikelet scales, and grain browning [22].

**Fig. 1:**
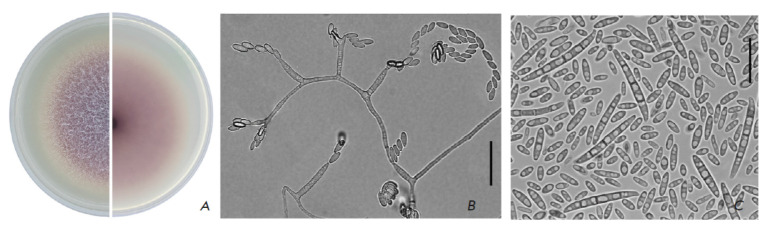
(A) – culture of F. proliferatum MFG 58486 (potato-sucrose agar, 7 days, 25°C, in the dark); (B) – microconidia on mono- and polyphyalides; (C) – microconidia and macroconidia (synthetic Nirenberg agar, 14 days, 25°C, in the dark). Scale bars = 20 μm

Due to the abundant formation of microconidia in false heads, short chains on mono- and polyphyalides, and macroconidia (*Fig. 1*), *F. proliferatum *is easily spread through the air and transferred by insects to new uninfected plants [23]. Like many other pathogens, it persists in seeds [14] and on plant debris in soil [24].

*F. proliferatum *has a teleomorphic stage characterized by the formation of perithecia containing ascospores on the substrate surface [25]. Sexual reproduction in heterothallic members of the FF species complex requires different sets of opposite matingtype genes, this characteristic determined by the MAT locus and its two idiomorphs, MAT1-1 and MAT1-2 [26]. A balanced effective population size, with roughly equal proportions of each mating type, is necessary for sexual reproduction in heterothallic species. A skewed distribution of mating types, however, can impair sexual sporulation and diminish intraspecific diversity [27].

Similar to other fungi of the FF species complex,* F. proliferatum *produces toxic secondary metabolites: FUM, BEA, MON, fusaproliferin, fusarins, fusaric acid, and others, which can accumulate in grain and pose a health hazard to its consumers [28]. A reliable relationship between *F. proliferatum *infection of wheat grain and the amount of FUM detected in it has been established [29, 30]. A summary of the current data on mycotoxin contamination in various cereal grains reveals that wheat and barley exhibit lower levels of fumonisin accumulation [31, 32, 33] compared to maize, which frequently displays significantly higher amounts [34, 35]. The mycotoxin amounts produced by *F. proliferatum *strains of different substrate origin can vary significantly, and both active producers and non-toxigenic strains can be found among them [8, 28, 29, 36, 37, 38].

The objective of this research was the phylogenetic identification of *F. proliferatum *strains isolated from cereal crops and the subsequent *in vitro *determination of their ability for mycotoxin production.

## EXPERIMENTAL

***Fusarium *strains**

A choice of twelve fungal strains, identified morphologically as belonging to the FF species complex, was made from the pure culture collection maintained in the laboratory of mycology and phytopathology of VIZR (*Table 1*). All the strains were isolated from grain samples collected from different regions of the Russian Federation: six from wheat (*Triticum aestivum* L.), four from oats (*Avena sativa *L.), and two from maize (*Zea mays *L.).

**Table 1:** F. proliferatum strains included in the study

Strain number	Origin	Host plant	Year	GenBank accsession number		
				tef	tub	rpb2
MFG^*^58227	Krasnodarskiy kray	wheat	2009	MW811114	OK000500	OK000527
MFG 58471	Krasnodarskiy kray	wheat	2012	MW811115	OK000501	OK000528
MFG 58486	Krasnodarskiy kray	wheat	2012	MW811117	OK000503	OK000530
MFG 59046	Krasnodarskiy kray	wheat	2016	MW811122	OK000508	OK000535
MFG 60309	Krasnodarskiy kray	wheat	2017	MW811125	OK000513	OK000540
MFG 60803	Amur region	wheat	2019	MW811134	OK000522	OK000549
MFG 58589	Leningrad region	oats	2013	MW811118	OK000504	OK000531
MFG 58590	Primorsky Krai	oats	2013	MW811119	OK000505	OK000532
MFG 92501	Leningrad region	oats	2007	MW811135	OK000524	OK000551
MFG 58667	Nizhny Novgorod region	oats	2014	MW811121	OK000507	OK000534
MFG 58484	Voronezh region	maize	2012	MW811116	OK000502	OK000529
MFG 58603	Lipetsk region	maize	2012	MW811120	OK000506	OK000533

^*^Note. MFG – the culture collection of the laboratory of mycology and phytopathology of VIZR, St. Petersburg, Russia.

**Molecular and genetic analysis**

Potato-sucrose agar (PSA) was used as the growth medium for all fungal strains. Cultivation occurred within a KBW 400 thermostat (Binder, Germany) at 25°C for 7 days. Fungal DNA was isolated from the mycelium via a standard protocol employing a 2% cetyltrimethylammonium bromide/chloroform solution.

The *tef*, *tub*, and *rpb2 *gene fragments were amplified using the primers EF1/EF2, T1/T2, and fRPB2-5F/fRPB2-7Cr [39]. The resulting fragments were sequenced by the Sanger sequencing method on an ABIPrism 3500 sequencer (Applied Biosystems – Hitachi, Japan) using the BigDye Terminator v3.1 Cycle Sequencing Kit (ABI, USA). The consensus nucleotide sequences were aligned in the Vector NTI Advance 10 program (Thermo Fisher Scientific, USA) and deposited in the NCBI GenBank database (*Table 1*).

The phylogenetic analysis involved nucleotide sequences from representative *Fusarium *strains from the collections of the Agricultural Research Service Cultural Collection (NRRL, USA), Westerdijk Institute for Fungal Biodiversity (CBS, The Netherlands), and other collections (*Table 2*). The phylogenetic relationships among taxa were evaluated by the ML method using the program IQ-TREE 2 v.2.1.3. Optimal nucleotide substitution modeling for maximum likelihood (ML) tree inference was achieved using TIM2e+R2, as determined by IQ-TREE 2 v.2.1.3. A bootstrap analysis (1 000 replicates) was conducted to evaluate the robustness of the phylogenetic tree topology. The BP values were calculated using MrBayes version 3.2.1, implemented on the Armadillo 1.1 platform.

**Table 2:** Structures of

Species	Strain number in the collection*	Origin	Substrate	Year	GenBank accsession number		
					tef	tub	rpb2
F. acutatum	CBS 402.97 T	India		1995	MW402125	MW402323	MW402768
F. acutatum	NRRL 13308	India		1985	AF160276	MW402348	MN193883
F. agapanthi	NRRL 54463 T	Australia	Agapanthus sp.	2010	KU900630	KU900635	KU900625
F. agapanthi	NRRL 54464	Australia	Agapanthus sp.	2010	MN193856	KU900637	KU900627
F. aglaonematis	ZHKUCC 22-0077 T	China	Aglaonema modestum, stem	2020	ON330437	ON330440	ON330443
F. aglaonematis	ZHKUCC 22-0078	China	Aglaonema modestum, stem	2020	ON330438	ON330441	ON330444
F. anthophilum	CBS 119859	New Zealand	Cymbidium sp., leaves		MN533991	MN534092	MN534233
F. anthophilum	CBS 222.76 T	Germany	Euphorbia pulcherrima, stem		MW402114	MW402312	MW402811
F. concentricum	CBS 450.97 T	Costa Rica	Musa sapientum, fruit	1983	AF160282	MW402334	JF741086
F. concentricum	CBS 453.97	Guatemala	Musa sapientum	1996	MN533998	MN534123	MN534264
F. elaeagni	LC 13627 T	China	Elaeagnus pungens	2017	MW580466	MW533748	MW474412
F. elaeagni	LC 13629	China	Elaeagnus pungens	2017	MW580468	MW533750	MW474414
F. erosum	LC 15877 T	China	maize, stem	2021	OQ126066	OQ126321	OQ126518
F. erosum	LC 18581	China	maize, cob	2021	OQ126067	OQ126320	OQ126519
F. fujikuroi	CBS 221.76 T	Taiwan	Oryza sativa, stem	1973	MN534010	MN534130	KU604255
F. fujikuroi	CBS 257.52	Japan	Oryza sativa, seedling	1947	MW402119	MW402317	MW402812
F. globosum	CBS 428.97 T	South Africa	Zea mays, seed	1992	KF466417	MN534124	KF466406
F. globosum	CBS 120992	South Africa	Zea mays, seed	1992	MW401998	MW402198	MW402788
F. hechiense	LC 13644 T	China	Musa nana	2017	MW580494	MW533773	MW474440
F. hechiense	LC 13646	China	Musa nana	2017	MW580496	MW533775	MW474442
F. lumajangense	InaCCF 872 T	Indonesia	Musa acuminata, stem	2014	LS479441	LS479433	LS479850
F. lumajangense	InaCCF 993	Indonesia	Musa acuminata, stem	2014	LS479442	LS479434	LS479851
F. mangiferae	CBS 120994 T	Israel	Mangifera indica	1993	MN534017	MN534128	MN534271
F. mangiferae	NRRL 25226	India	Mangifera indica		AF160281	U61561	HM068353
F. nirenbergiae	CBS 744.97	USA	Pseudotsuga menziesii	1994	AF160312	U34424	LT575065
F. nygamai	NRRL 13448 T	Australia	Sorghum bicolor	1980	AF160273	U34426	EF470114
F. nygamai	CBS 834.85	India	Cajanus cajan		MW402154	MW402355	MW402821
F. panlongense	LC 13656 T	China	Musa nana	2017	MW580510	MW533789	MW474456
F. panlongense	MUCL 55950	China	Musa sp.	2012	LT574905	LT575070	LT574986
F. proliferatum	NRRL 22944	Germany	Cymbidium sp.	1994	AF160280	U34416	JX171617
F. proliferatum	ITEM 2287	Italy			LT841245	LT841243	LT841252
F. proliferatum	NRRL 31071	USA	wheat	2001	AF291058	AF291055	
F. proliferatum	NRRL 32155	India	Cicer arietinum		FJ538242		
F. proliferatum	CBS 131570	Iran	wheat		JX118976		JX162521
F. sacchari	CBS 223.76 T	India	Saccharum officinarum	1975	MW402115	MW402313	JX171580
F. sacchari	CBS 131372	Australia	Oryzae australiensis, stem	2009	MN534033	MN534134	MN534293
F. sanyaense	LC 15882 T	China	maize, stem	2021	OQ126093	OQ126322	OQ126547
F. sanyaense	LC 18540	China	maize, stem	2021	OQ126095	OQ126308	OQ126549
F. siculi	CBS 142222 T	Italy	Citrus sinensis	2015	LT746214	LT746346	LT746327
F. siculi	CPC 27189	Italy	Citrus sinensis		LT746215	LT746347	LT746328
F. sterilihyposum	NRRL 53991	Brazil	Mangifera indica	2009	GU737413	GU737305	
F. sterilihyposum	NRRL 53997	Brazil	Mangifera indica	2009	GU737414	GU737306	
F. subglutinans	CBS 536.95				MW402139	MW402339	
F. subglutinans	CBS 136481	Italy	human blood		MW402059	MW402258	MW402748
F. verticillioides	NRRL 22172	Germany	maize	1992	AF160262	U34413	EF470122
F. verticillioides	CBS 531.95		Zea mays		MW402136	MW402336	MW402771
F. xylaroides	NRRL 25486 T	Côte d’Ivoire	Coffea sp., stem	1951	AY707136	AY707118	JX171630
F. xylaroides	CBS 749.79	Guinea	Coffea robusta	1963	MN534049	MN534143	MN534259

^*^Note. Acronyms of the culture collections: CBS – the Westerdijk Institute for Fungal Biodiversity (Utrecht, The Netherlands); InaCCF – the Indonesian Biology Research Center (Cibinong, Indonesia); ITEM – the Institute of Science of Food Production (Bari, Italy); LC – the laboratory of Dr. Lei Cai, Institute of Microbiology, Chinese Academy of Sciences (Beijing, China); MUCL – the Laboratory of Mycology, Université Catholique de Louvain (Ottigny-Louvain-la-Neuve, Belgium); NRRL – the Agricultural Research Service Cultural Collection (Peoria, USA); ZHKUCC – the Zhongkai University of Agriculture and Engineering (Guangzhou, China); T – type strain.

The mating type of the strains was identified by allele-specific PCR. The primers Gfmat1a/Gfmat1b (MAT1-1) and Gfmat1c/Gfmat1d (MAT1-2), designed for the FF species complex, were employed in accordance with the protocol in [40], but the annealing temperature was changed to 55°C. The fragment sizes corresponding to the MAT1-1 and MAT1-2 alleles were 200 and 800 bp, respectively.

**Mycotoxin analysis**

A mixture of twenty grams of rice grains and twelve milliliters of water contained within 250 mL glass vessels underwent autoclaving at 121°C for forty minutes. Following the autoclaving, the rice grains were cooled and inoculated with two 5 mm diameter disks cut from fungal cultures grown on PSA. Uninoculated grains served as the control. A two-week incubation period in the dark at 25°C was implemented, with daily shaking of the flasks. The samples were dried at 55°C for 24 h, then ground using a laboratory mill (IKA, Germany) at 25 000 rpm for one minute, and subsequently stored at -20°C.

HPLC-MS/MS analysis was used to determine the profile of secondary toxic metabolites [41]. Five grams of rice flour were combined with 20 milliliters of extraction solvent (acetonitrile/water/acetic acid, 79 : 20 : 1). Secondary metabolites detection and quantification were conducted using an AB SCIEX Triple Quad™ 5500 MS/MS system (Applied Biosystems, USA), incorporating a TurboV electrospray ionization source (SCIEX, USA) and an Agilent Infinity 1290 series microwave analysis system (Agilent, USA). Chromatographic separation was achieved using a Phenomenex (USA) Gemini C18 column (150 × 4.6 mm) at a temperature of 25°C.

The content of FB1, FB2, FB3, BEA, and MON were analyzed in the extracts. Mycotoxin recovery rates ranged from 79% to 105%. Mycotoxin quantification was achieved through a comparative analysis of peak areas against the calibration curves generated from standard solutions (Romer Labs Diagnostic GmbH, Austria). The limits of quantification for BEA and MON were 1.9 and 3.1 μg/kg, respectively; FB1, FB2, and FB3 displayed limits of 8.7, 3.2, and 3.2 μg/kg, respectively.

**Statistical analysis**

Statistical computations were performed with the aid of Microsoft Excel 2010 and Minitab 17.0.

## RESULTS AND DISCUSSION

**Molecular and genetic characterization of the strains**

**Fig. 2:**
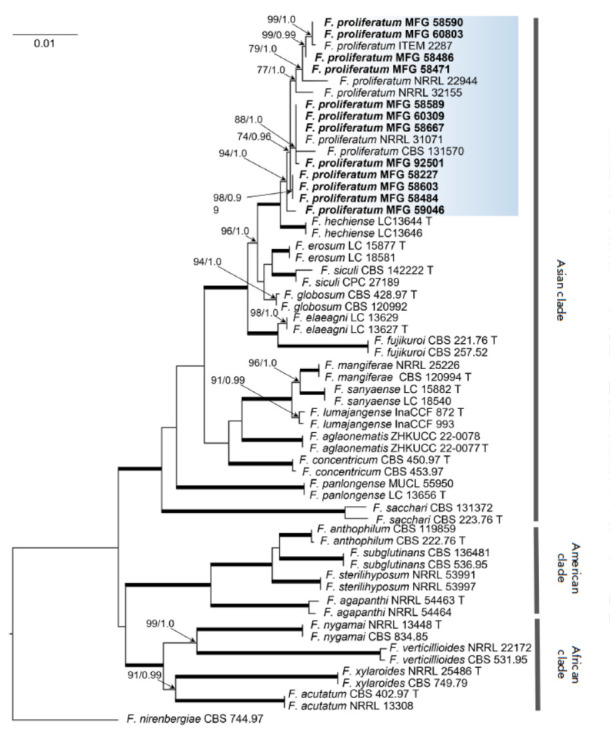
Dendrogram of phylogenetic similarity of Fusarium spp. based on combined nucleotide sequences of the tef, tub, and rpb2 gene fragments by the ML method. Nodes show bootstrap support values ( < 70%) in the ML analysis, as well as BP values ( < 0.95). The thickening of lines signifies support at the 100/1.0 ML/BP level. Strains within the study, obtained from the MFG collection, are denoted in bold. F. nirenbergiae strain CBS 744.97 was designated as the outgroup

The phylogenetic analysis included the combined sequences (1 913 bp) of three loci: *tef *– 615 bp, *tub *– 473 bp, and *rpb2 *– 825 bp, with 154 bp (25.0%), 70 bp (14.8%), and 141 bp (17.1%) informative sites, respectively. All the twelve strains were clustered to a sep- arate bootstrap-supported clade, ML/BP 94/1.0, also including five reference strains of *F. proliferatum* (*Fig. 2*). The *F. proliferatum *clade was distributed among the Asian group of FF species complex, and the topology of phylogenetic trees constructed by different methods was similar and consistent with the one reconstructed previously [1]. The resulting phylogenetic tree demonstrates significant genetic diversity within the *F. proliferatum *strains. The clades contained both the analyzed and reference strains, exhibiting no correlation between grouping and geographic or substrate source. Previous studies [8, 42, 43] have also observed a comparable categorization of *F. proliferatum *due to the substantial intraspecific variability of the species, irrespective of strain origin.

Specific PCR analysis demonstrated the presence of only one idiomorph at the MAT locus per *F. proliferatum* strain genotype, yielding an 8 : 4 ratio of MAT1-1 to MAT1-2 idiomorphs among the strains examined. The MAT locus is represented exclusively by the MAT1-2 idiomorph in the strains from maize and exclusively by the MAT1-1 idiomorph in the strains from oat. The MAT locus in the strains from wheat exhibited a 4 : 2 ratio of MAT1-1 to MAT1-2 alleles.

The disproportionate prevalence of alternative mating types within the *F. proliferatum *populations appears to correlate with a decreased frequency of sexual reproduction in the wild, consequently limiting genetic diversity. Furthermore, this impacts the pathogen’s capacity to adapt to fluctuating environmental conditions. The ratio of *F. proliferatum *strains isolated from cultivated plants with different idiomorphs at the MAT locus has been previously shown to vary [8, 42]. However, the *F. proliferatum *strains isolated from durum wheat grain in Argentina were characterized by an equal frequency of alternative alleles of the MAT locus, which allowed researchers to predict a high probability of detecting the sexual stage of the fungus in wheat fields [42].

**Profile of the mycotoxins produced by *F. proliferatum***

All five mycotoxins (BEA, MON, FB1, FB2, and FB3) were detected in extracts from rice grains inoculated by *F. proliferatum *strains. However, these were absent in the control

**Fig. 3:**
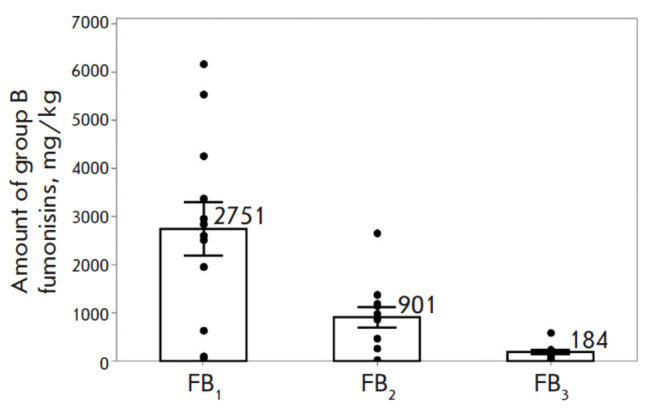
Fumonisins production by F. proliferatum strains (autoclaved rice, 25°C, 14 days, in the dark). Presented are the mean values and the confidence intervals at a significance level of p > 0.05. The dots indicate the values for individual strains

All strains exhibited significant FUM production ranging from 100 to 9 424 mg/kg. FB1 proved to be the predominant mycotoxin, amounting to 53–82% of total FUM. The mycotoxins FB2 and FB3 were found to be present in lower quantities, amounting to 9–28% and 2–39%, respectively. Among all the strains tested, MFG 58590 — isolated from oat grain originating in Primorsky Krai , Russia — produced the maximum amount of FUM. A marked reduction in total FB1, FB2, and FB3 was observed in the strains MFG 92501 and MFG 60803 (100 and 135 mg/kg, respectively), compared to the other strains (1 077–7 077 mg/kg) (*Fig. 3*).

The BEA production in all the *F. proliferatum* strains was similarly high, ranging between 64 and 455 mg/kg. The MON production proved substantially less than that of the four other mycotoxins, displaying variability from 12 to 6 565 µg/kg. The analysis of strain MFG 92501 indicated no presence of MON within its mycotoxin profile.

**Table 3:** Toxin-producing ability of F. proliferatum strains isolated from different cereal crops

Host plant (number of strains)	Mycotoxins^*^		
	FUM, mg/kg	BEA, mg/kg	MON, µg/kg
Wheat (6)	3470 ± 1008	307 ± 67	1690 ± 764
Oat (4)	4024 ± 1930	385 ± 43	260 ± 158
Maize (2)	3538; 5578	363; 158	1041; 6565

^*^Presented are the mean values and the confidence intervals at a significance level of p < 0.05.

The predominant FUM in the mycotoxin profile of* F. proliferatum *is FB1, a characteristic independent of strain substrate origin [12, 37, 38, 44]. Our study has not revealed any statistically significant correlation between strain substrate origin and mycotoxin production (*Table 3*). The growth and fumonisin production of *F. proliferatum *are known to be affected by a multitude of abiotic and biotic factors [45, 46, 47, 47]. The extensive host range of *F. proliferatum *demonstrates its considerable adaptive capacity, partly attributable to the synthesis of secondary metabolites. The ability to produce mycotoxins was found to be unrelated to the host plant from which *F. proliferatum *was isolated [23]. Infection of wheat with strains of this fungus isolated from different hosts resulted in the accumulation of FB1 and BEA in the grain [23], despite the fact that the strains initially differed in toxin- producing ability, but the detected amount of FB1 in infected wheat was much lower than that usually found in maize. The *F. proliferatum *strains isolated from maize grain were previously shown to possess a more variable FB1 production ability than strains isolated from wheat grain [36]. The function of FUM, specifically FB1, as a pathogenicity factor in *F. proliferatum* remains a subject of debate [48]. A cluster of genes (*FUM*) responsible for the biosynthesis of these mycotoxins has been identified in FUM producing* Fusarium *fungi [1, 11]. In contrast to *FUM19*, the genes *FUM1*, *FUM6*, *FUM8*, and *FUM21 *were demonstrated to be essential for FUM synthesis in the* F. proliferatum *strains. The deletion of these genes leads not only to the loss of the ability of fungus to synthesize these mycotoxins, but also to a decrease in its aggressiveness against the host plant [49]. At the same time, it was recently discovered that *F. proliferatum* strains isolated from garlic could produce FUM* in vitro *but did not necessarily produce them *in planta* [38]. Furthermore, fungal exposure to host plant metabolites during colonization may influence mycotoxin production and concentration [50]. Although* F. proliferatum *inhabits the mycobiota of Eurasian wheat, barley, and oat, elevated fumonisin amounts in their grains are atypical, contrasting with the common detection of beauvericin and the less frequent detection of moniliformin [30, 51, 52]. Presumably, wheat grain is a less suitable substrate for FUM accumulation than maize [23, 44].

## CONCLUSIONS

The phylogenetic study of *F. proliferatum *strains isolated from three cereal crops grown on the territory of the Russian Federation demonstrated significant intraspecific heterogeneity, independent of the geographical and substrate strain origin. Such an uneven distribution of *F. proliferatum *strains with differing mating types is likely to diminish the significance of sexual reproduction in the life cycle of this heterothallic fungus. In conjunction with environmental factors, the considerable mycotoxin production potential of *F. proliferatum *suggests a high risk of grain contamination, thus necessitating systematic monitoring.

## Abbreviations

FF: Fusarium fujikuroi
tef: the translation elongation factor 1-α gene
tub: β-tubilin gene
rpb2: second subunit gene of RNA polymerase II
ML (maximum likelihood): maximum likelihood method
BP (Bayesian probability): Bayesian posterior probability scores
MAT locus: mating type locus
HPLC-MS/MS: high-performance liquid chromatography coupled with a tandem mass spectrometry
FUM: group B fumonisins
FB1: fumonisin B1
FB2: fumonisin B2
FB3: fumonisin B3
BEA: beauvericin
MON: moniliformin
